# Ovine *HOXB13*: expanding the gene repertoire of sheep tail patterning and implications in genetic improvement

**DOI:** 10.1038/s42003-022-04199-7

**Published:** 2022-11-07

**Authors:** Peter Kalds, Shuhong Huang, Yulin Chen, Xiaolong Wang

**Affiliations:** 1 International Joint Agriculture Research Center for Animal Bio-breeding, Ministry of Agriculture and Rural Affairs/Key Laboratory of Animal Genetics, Breeding and Reproduction of Shaanxi Province, College of Animal Science and Technology, Northwest A&F University, Yangling, 712100 China; 2 Department of Animal and Poultry Production, Faculty of Environmental Agricultural Sciences, Arish University, El-Arish, 45511 Egypt

**Keywords:** Animal breeding, Evolutionary genetics

## Abstract

Recent findings implicating *homeobox B13* (*HOXB13*) as a regulator of sheep tail length and their impact on sheep breeding via selection-based strategies and molecular genetics-based tools are discussed.

## Teaser

Understanding the genetic basis of sheep tails is of great importance to improve sheep production and welfare. In this Comment, we discussed recent findings showing that *homeobox B13* (*HOXB13*) is a regulator of the tail length in sheep. The revelation of the *HOXB13* gene adds novel insights into the genetic determinism of the sheep tail phenotype. This new genetic highlight will lead to further advances in sheep breeding using selection-based strategies and molecular genetics-based tools.

## A brief background: piecing together the genetic structure of sheep tail formation

**Fig. 1:**
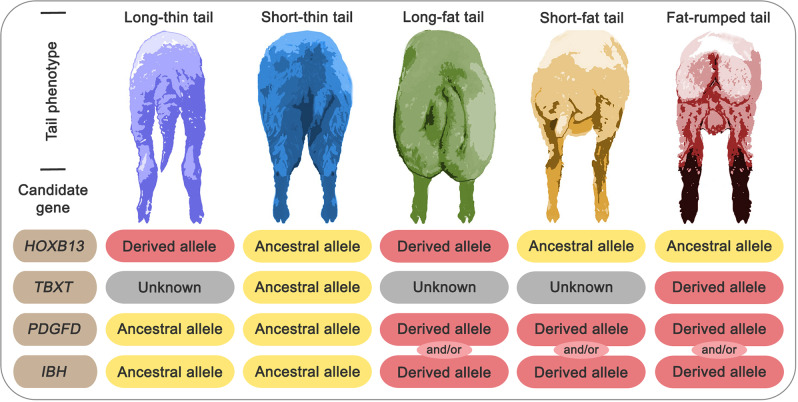
A proposed hypothesis of the genetic structure of sheep tail patterning based on the currently known, significant four genomic signatures. The upper panel shows the main five sheep tail phenotypic patterns, including the long-thin tail (e.g., Merino sheep), short-thin tail (e.g., Tibetan sheep), long-fat tail (e.g., Chinese Large-tailed Han sheep), short-fat tail (e.g., Chinese Hu sheep), and fat-rumped tail (e.g., Kazakh sheep). Note: Sheep tails characterized by intermediate tail length and level of fat deposition are shown in some breeds. This intermediate pattern of the fat tail can be categorized between the long-fat tail and the short-fat tail (shown in the third and fourth tail phenotypic patterns in the upper panel). The lower panel shows the main candidate genes/regions of the sheep tail phenotype (in brown boxes). These include two genes linked with the sheep tail length [*HOXB13* (refs. ^21,23^) and *TBXT* (refs. ^16–18^)] and two genes/regions linked with the fat-tail phenotype [*PDGFD* (refs. ^1,11,12^) and the intergenic region between *BMP2* and *HAO1*, referred to as the *IBH* region^13–15^]. Yellow, red, and gray boxes indicate ancestral allele, derived allele, and currently unknown functional role, respectively. The icons of sheep tails used to construct this figure were adapted from Kalds et al.^3^.

The tail phenotype, an obvious body segment, has been described as a significantly divergent trait in sheep^1^, which is due to the wide phenotypic diversity of sheep tails among different breeds located within various geographical and environmental regions^2^. Several factors influence the sheep tail phenotype, including length, fat deposition level, and directionality. The overall tail length is controlled by the number and length of caudal vertebrae. On the other hand, tail fat deposition is controlled by the size and number of adipocytes. Several tail phenotypic patterns have been shown in sheep, including the thin-long tail, thin-short tail, fat-short tail, fat-long tail, and fat-rumped tail (Fig. 1, upper panel). Studies have been performed to understand the mechanistic determinism of sheep tails based on genetic basis, due to the association of sheep tails with several raising-related and welfare issues (see reviews by refs. ^3–6^). Of these issues, tail docking, as a painful practice, is the most critical^5,6^. Revealing the genetic determinism of sheep tails will help to direct the selection towards the desired phenotypes^7,8^ and provide access to new biotechnological tools to directly introduce the desired variant(s) into the targeted breed^9,10^. Recently, three genes/regions were reported to be significantly associated with the sheep tail phenotype. These include the *platelet derived growth factor D* (*PDGFD*) gene^1,11,12^ and the intergenic region between the *bone morphogenetic protein 2* (*BMP2*) and *hydroxyacid oxidase 1* (*HAO1*) genes, referred to as the *IBH* region^13–15^ linked with the fat-tail phenotype, and the *T-box transcription factor T* (*TBXT*) gene^16–18^ linked with variations in caudal vertebrae. Recent studies highlighted a new gene, *HOXB13*, which is highly associated with the sheep tail length^19–23^ (Table 1). Of these studies, two independent efforts, one recently published in *Communications Biology*^23^ and the other published as a preprint^21^, strongly highlighted a structural variation (SV) in the form of a short insertion associated with the long-tail phenotype, adding a new critical member to the gene repertoire of the sheep tail configuration.

**Table 1:** A summary of genomic studies that highlighted the *HOXB13* gene associated with the tail phenotype in the ovine genome.

Reference	Breed(s) and tail phenotype(s)^*^	Origin	Genotyping approach	Ovine reference genome
Ahbara et al. 2019 (ref.^19^)	Fat-rumped (Kefis, Adane, and Arabo). Short fat-tailed (Molale-Menz). Long fat-tailed (Bonga, Gesses, Kido, Doyogena, Shubi-Gemo, and Loya)	Ethiopia	Ovine 50 K SNP BeadChip (Illumina)	Oar_v3.1
	Long thin-tailed (Hammari and Kabashi)	Sudan		
Manzari et al. 2019 (ref.^20^)	Fat-tailed (Baluchi and Lori-Bakhtiari). Thin-tailed (Zel)	Iran	Ovine 50 K SNP BeadChip (Illumina)	Oar_v3.1
Li et al. 2022 (ref.^21^)	Long thin-tailed (Suffolk, Dorset, Texel, Charollais, Merino, and Romney). Rat-tailed (East Friesian)	Europe	Long-read PacBio HiFi sequencing	ARS-UI_Ramb_v2.0
	Long thin-tailed (White Dorper)	Africa		
	Fat-tailed (Kermani)	Middle east (Iran)		
	Short fat-tailed (Ujumqin). Fat-rumped (Kazakh)	East Asia (Northern China)		
	Short thin-tailed (Tibetan)	East Asia (Qinghai-Tibetan Plateau)		
	Short thin-tailed (Yunnan)	East Asia (Yunnan-Kweichow Plateau)		
Ahbara et al. 2022 (ref.^22^)	Fat-rumped (Kefis, Segentu, Adane, and Arabo). Short fat-tailed (Gafera-Washera and Molale-Menz). Long fat-tailed (Bonga, Gesses, Kido, Doyogena, Shubi-Gemo, and Loya)	Ethiopia	Paired-end sequencing (Illumina)	Oar_v3.1
	Long thin-tailed (Hammari and Kabashi)	Sudan		
	Long fat-tailed (shorter caudal vertebrae length; Barberine)	Libya		
Lagler et al. 2022 (ref.^23^)	Long thin-tailed (Merinolandschaf)	German	Ovine 50 K SNP BeadChip (Illumina)	Oar_v4.0

*In some of the indicated studies, further validations of potential variants were performed using expanded number of individuals from different populations.

## *HOXB13* as a controller of long and short tails: variant determination in the ovine context

In recent studies^21–23^, the *HOXB13* gene was first highlighted to be significantly associated with the sheep tail phenotype in a genomic comparison that included Ethiopian fat-rumped and short fat-tailed sheep breeds *vs*. Ethiopian long fat-tailed and Sudanese long thin-tailed sheep breeds^22^. In this genomic analysis, a haplotype specific to Ethiopian long fat-tailed and Sudanese long thin-tailed sheep breeds was highlighted (top variant: rs428121282; chr11:37,338,422; Oar_v3.1)^22^. Subsequently, by applying the long-read PacBio sequencing and verifying the frequency spectrum of SVs in an expanded dataset, the most differentiated SV was detected as insertion of 169 bp (chr11:37,525,005; ARS-UI_Ramb_v2.0) close to the 5’ untranslated region (5’UTR) of the *HOXB13* gene^21^. The prevalence of this potential insertion was observed in long-tailed sheep breeds. The long-read sequencing approach could also reveal previously undetected SVs in sheep tail-related genes. These include six highly differentiated SVs in the *IBH* selective region (the largest event was a 7728-bp insertion) and three SVs in the *PDGFD* selective region (the largest one was an 867-bp insertion)^21^. More recently, in the Merinolandschaf breed, the same variant was highlighted as a 167-bp insertion in the promotor region of *HOXB13* with a completely linked nonsynonymous mutation in the first exon of *HOXB13* located 132 bp downstream of the insertion (rs413316737; chr11:37,290,361; OAR_v4.0; c.C23G)^23^. This 167-bp insertion was flanked by 14-bp direct repeats (CTGCCAGCGATTTA) on both sides, which suggests the potential that this insertion is a short interspersed nuclear element (SINE)^23^. Differences in the length and position of this variant were indicated as a result of sequencing error due to the presence of a long ‘T’ base homopolymer in the detected SINE repeat element^23^. Future investigations may particularly identify the exact role of the *HOXB13* insertion variant. The synchronized discovery of this critical variant by these two independent studies using different breeds supports its high potential causality in the formation of sheep tail length.

## Long or short: the influence of the ovine *HOXB13* variant on the tail phenotype and the nature of gene expression

The influence of this insertion on the tail phenotype was also highlighted. In 211 individuals from an F2 population of backcrossing East Friesian sheep (♂) with [Hu sheep (♀) × East Friesian sheep (♂)], the tail length of the homologous carriers of the insertion was significantly longer than that of the heterozygous carriers by 2.77 cm^21^. A similar finding was also observed in the Merinolandschaf sheep, where tail lengths of homozygous carriers, heterozygous carriers, and non-carriers of the insertion were 31.5, 25.7, and 24.1 cm, respectively^23^. The expression of *HOXB13* with and without the detected insertion variant was investigated using a luciferase reporter assay^21^. Compared to the wild-type sequence, the insertion decreased the luciferase activity by 10-fold^21^, indicating that the reduced *HOXB13* transcriptional level was linked with the long-tail phenotype. Interestingly, such results were previously proven using mouse models. By applying genetic manipulation techniques to induce *HOXB13* gene loss- and gain-of-function activities in mice, *HOXB13* deficiency causes tail overgrowth and an increased number of caudal vertebrae, whereas *HOXB13* overexpression results in prematurely truncated tails and transgenic *HOXB13*-overexpressing mice have shorter tails^24–26^. These novel findings in sheep and previous investigations in mice support the involvement of the *HOXB13* gene in patterning tail length in mammals.

## A proposed hypothesis of the sheep tail genetic structure: looking for an elucidation

With the emergence of *HOXB13*, as member of the gene repertoire of the sheep tail configuration, a clearer picture regarding the genetic structure of sheep tails could be drawn. The wild ancestor of sheep, Asiatic mouflon (*Ovis orientalis*), shows a short thin-tail phenotype^27^, which suggests that sheep breeds with divergent tail phenotypes, e.g., long thin and long fat phenotypes, emerged later (~5000 years ago)^28^. The first domesticated sheep were initially used as a source of food^29^. After several millennia, the production and processing of wool have emerged, leading to the selection and a worldwide spread of wool sheep (e.g., Merino and Merino-derived breeds). These wool-producing breeds are characterized by long tails and there is a common occurrence of fine wool and long tail, rising the hypothesis that these two traits are genetically linked or emerged as a result of the same artificial selector^23,30^. Fat-tailed sheep are known for storing fat in their tails as an energy reserve, which is considered an adaptive response to the harsh environment and food scarcity^28^. Additionally, ancient breeders selected them for their adaptability and as a traditional cooking fat and energy source for human consumption. Therefore, selection for the fat-tailed phenotype could have been initiated and promoted by extreme climatic conditions and/or artificial selectors’ preferences^31^. Particularly, by combining single nucleotide polymorphisms (SNPs) of the male-specific region of the Y chromosome with mitochondrial DNA (mtDNA) variations and whole-genome sequences of rams from the worldwide sheep population, Deng et al.^31^ reported that (i) the first domestic (hair-coated) sheep spread ~10,500 years BP (9000–11,800) from the Fertile Crescent (the Near Eastern domestication center); (ii) the selection for secondary products (e.g., wool) triggered the second expansion of sheep populations ~8000–7000 years BP, most likely from Southwest Asia at first; and (iii) a later (third) spread was likely associated with the expansion event of fat-tail sheep ~3400 years BP (1700–5300) from the Middle East to Northern Africa, Central and Eastern Asia, and the eastern edge of Europe.

The expanded patterns of tails at the caudal vertebrae and fat deposition levels require active growth factors. It is not surprising that all the detected genes for the sheep tail phenotype (*PDGFD*, *BMP2*, *TBXT*, and *HOXB13*) have a relationship with cancer development and progression^32–35^. The above-mentioned four genomic signals were detected to influence the sheep tail phenotype at both levels, the caudal vertebrae number (*TBXT* and *HOXB13*)^16–23^ and the level of tail fat deposition (*PDGFD* and *BMP2*)^1,11–15,36^. Thus, making the genetic framework of the sheep tail phenotype clearer to be inferred. Here, based on the potential function and variant association of these four genes/regions, we hypothesize a potential genetic structure of the five main sheep tail phenotypes (Fig. 1). It is expected that sheep with the short thin-tail phenotype carry ancestral alleles of the four genomic signatures. Fat-rumped sheep with very few numbers of caudal vertebrae and a high level of tail fat deposition were shown to carry a derived allele in the *TBXT* gene (Chr8:87,804,589 G > T; Oar_v3.1; c.G334T)^16,17^ that was functionally validated using genome editing^18^ and it is expected to carry derived alleles in one or both of the genomic signatures linked with tail fat deposition (*PDGFD* and/or *BMP2*)^1,11–15^. Long thin-tailed sheep are expected to carry a derived allele in the *HOXB13* gene^21,23^ with ancestral alleles for the two fat deposition-related genomic signatures. Long fat-tailed sheep are expected to carry derived alleles in the *HOXB13* gene and one or both of the fat deposition-related genomic signatures. The same situation was observed in the case of the short fat-tailed sheep, but potentially with an ancestral *HOXB13* allele. In long thin-tailed, long fat-tailed, and short fat-tailed sheep, the potential role of the *TBXT* gene in tail patterning is unknown.

Collectively, in terms of tail length, both genes, *HOXB13* and *TBXT*, have been shown to play functional roles; however, in different directions. According to the current research, mutations in *HOXB13* (refs. ^21,23,24,26^) and *TBXT* (refs. ^16–18,37,38^) have been shown to increase and reduce the tail length, respectively. Although the obvious roles of the currently known genomic signatures, other causal variants explaining a wider range of phenotypic variance are likely still unknown.

## Concluding remarks: towards a customized sheep tail phenotype

Sequencing and annotating livestock genomes are important for improving breeding based on known genetic background^39,40^. Genetic intervention could solve issues related to production, fertility, environmental adaptation, and animal welfare. In sheep, there are two main ways to eliminate the undesired long and/or fat tail phenotypes, including tail docking and crossbreeding. Tail docking is a painful procedure implicated in animal welfare issues. On the other hand, crossbreeding can generate a proportional reduction in tail length. However, a potential loss of previously achieved breeding progress of economically important traits is highly expected. Thus, the accumulated genomic knowledge regarding the sheep tail phenotype could help improve sheep breeding without compromising other desired traits. The revelation of causative variants, such as *HOXB13* variants, could provide solutions through the direct gene-assisted selection of the desired short tail ancestral alleles ‘back to the roots^23^’. In addition, potential biotechnological intervention using modern genome-editing tools could be used to install desired variants without compromising other previously selected genome content. Potential *HOXB13* variant(s) might be solely helpful to shorten the tail length in thin long-tailed sheep breeds. In long fat-tailed breeds, it can play a role in combination with tail fat deposition-related gene(s) to generate a shorter tail with a reduced level of fat deposition.

Significant SVs have been discovered due to emerging long-read sequencing technologies^41–43^. In sheep, SVs have been shown to play major roles in phenotypic variations. These include, e.g., SVs in *relaxin family peptide receptor 2* (*RXFP2*) (refs. ^44,45^) and *interferon regulatory factor 2 binding protein 2* (*IRF2BP2*) (refs. ^46,47^), are linked with horn and wool phenotypes, respectively. Further application of long-read sequencing technologies is needed to interrogate the ovine genome. These genomic advances can ameliorate the sheep industry to face future challenges, including climate change, the increasing human population, and the ever-increasing demand for animal products. This could be possible by applying genetic-based strategies, including direct gene-assisted selection and the application of modern genome-editing tools.

## Supplementary information

nr-reporting-summary
